# Quality of life and satisfaction of students with auriculotherapy in the covid-19 pandemic: a quasi-experimental study

**DOI:** 10.1590/0034-7167-2022-0522

**Published:** 2023-02-03

**Authors:** Caroline de Castro Moura, Bárbara Guimarães Lourenço, Bruna de Oliveira Alves, Bianca Bacelar de Assis, Luana Vieira Toledo, Ludmila de Oliveira Ruela, Tânia Couto Machado Chianca

**Affiliations:** I Universidade Federal de Viçosa. Viçosa, Minas Gerais, Brazil; II Universidade Federal de Minas Gerais. Belo Horizonte, Minas Gerais, Brazil; III Centro Universitário de Lavras. Lavras, Minas Gerais, Brazil

**Keywords:** Students, Universities, COVID-19, Quality of Life, Auriculotherapy., Estudiantes, Universidades, COVID-19, Calidad de Vida, Auriculoterapia., Estudantes, Universidades, COVID-19, Qualidade de Vida, Auriculoterapia.

## Abstract

**Objectives:**

to evaluate the quality of life before and after the application of auriculotherapy and the satisfaction of university students with the treatment during the covid-19 pandemic.

**Methods:**

quasi-experimental study conducted with 44 students in a University Health Center. The intervention consisted of ten sessions of auriculotherapy focusing on emotional changes with quality of life assessment before and after treatment. The study also investigated the satisfaction concerning the intervention.

**Results:**

predominated among the students: women, from health courses, in use of psychotropic drugs and complaining of emotional changes. There was a statistically significant increase in all domains of quality of life, and students were satisfied with the treatment.

**Conclusions:**

auriculotherapy improved the quality of life of university students during the covid-19 pandemic, and the level of satisfaction with the treatment was high.

## RESUMO

**Objetivos:**

avaliar a qualidade de vida antes e depois da aplicação da auriculoterapia e a satisfação de estudantes universitários com o tratamento durante a pandemia de covid-19.

**Métodos:**

estudo quase experimental, realizado com 44 estudantes em um centro de saúde de uma universidade. A intervenção consistiu em dez sessões de auriculoterapia, com foco nas alterações emocionais, e a avaliação da qualidade de vida ocorreu antes e depois do tratamento. A satisfação com a intervenção também foi investigada.

**Resultados:**

predominaram entre os estudantes: mulheres, de cursos da área da saúde, em uso de psicotrópicos e com queixa de alterações emocionais. Houve aumento estatisticamente significativo em todos os domínios da qualidade de vida, e os estudantes ficaram satisfeitos com o tratamento.

**Conclusões:**

a auriculoterapia foi capaz de melhorar a qualidade de vida de estudantes universitários durante a pandemia de covid-19, e o nível de satisfação com o tratamento foi alto.

## RESUMEN

**Objetivos:**

evaluar la calidad de vida antes y después de la aplicación de auriculoterapia y satisfacción de estudiantes universitarios con el tratamiento durante la pandemia de covid-19.

**Métodos:**

estudio cuasi experimental, realizado con 44 estudiantes en centro de salud de una universidad. La intervención constituyó en diez sesiones de auriculoterapia, enfocado en las altercaciones emocionales, y la evaluación de la calidad de vida pasó antes y después del tratamiento. La satisfacción con la intervención también fue investigada.

**Resultados:**

predominaron entre los estudiantes: mujeres, de cursos del área de la salud, en uso de psicotrópicos y con queja de alteraciones emocionales. Hubo aumento estadísticamente significante en todos los dominios de la calidad de vida, y los estudiantes quedaron satisfechos con el tratamiento.

**Conclusiones:**

la auriculoterapia fue capaz de mejorar la calidad de vida de estudiantes universitarios durante la pandemia de covid-19, y el nivel de satisfacción con el tratamiento fue alto.

## INTRODUCTION

The years of 2020 to 2022 were marked by the Covid-19 pandemic, a disease of great transmissibility and clinical severity^(1)^. Among the numerous consequences of the pandemic, the need for social distancing stands out as one of the main conducts to prevent the spread of the virus^(2)^. This need led to the suspension of academic activities in universities around the world. Although necessary, prolonged social distancing caused physical, psychological, social, and economic problems for university students, which considerably impacted their quality of life greatly.

Quality of life can be defined as the individual’s perception of their position in life in the context of the culture and value system in which they live and regarding their goals, expectations, standards, and concerns^(3)^. It is a broad concept that covers the interrelation with the environment, physical and psychological aspects, level of independence, social relationships, and personal beliefs^(4)^.

During epidemics, the effects on people’s mental health are usually more severe than the number of infected, and it can be potentiated in case of an infection of pandemic characteristics^(5)^. In this way, mental symptoms can be triggered in healthy people, intensified in those with preceding mental comorbidities, and lead to increased suicide rates^(6)^. Then, considering that the quality of life was negatively affected in the pandemic context, the importance of implementing actions that can assist in the psychic and emotional management of university students is emphasized so that they can perform academic, social, and personal assignments in a satisfactory and healthy way.

Auriculotherapy is a therapeutic resource recently used to improve quality of life. Its use is intended for people with chronic pain^(7)^ and nursing professionals with high levels of stress^(8)^. It is an intervention used to relieve psychosomatic and physical dysfunctions through the stimulation of specific points in the ears that triggers the release of biomolecules, such as neurotransmitters and endogenous peptides at the central level, contributing to the action of the technique^(9)^. Due to the low cost, the possibility of mass treatment, and minimal or absent side effects, the great interest, acceptance, and projection achieved by this intervention worldwide are undeniable.

In the university context, a study points out that auriculotherapy, associated or not with other treatments, can contribute to improving the quality of life of students, improving academic performance and reducing school dropout, so it should be considered by training institutions for mental health promotion^(10)^. However, studies related to the pandemic context are scarce, pointing to a little explored scientific reality. In addition, with the return of face-to-face academic activities, after two years of absence, there was an exponential increase in complaints of emotional disorders experienced by students, especially anxiety, stress and depression, which reaffirms the need to implement strategies for the prevention and remediation of traumatic episodes resulting from the Covid-19 pandemic^(11)^.

To subsidize the implementation of this therapy in the psychosocial health care services of university students, it is also necessary to evaluate their degree of satisfaction in terms of feelings about the treatment (if the positive characteristics outweigh the negative ones)^(12)^. In addition to making the participant the protagonist of their care plan, the result of this evaluation can generate indicators that will favor strategies to promote the well-being and quality of life of the university community.

## OBJECTIVES

To evaluate the quality of life before and after the application of auriculotherapy and the satisfaction of university students with the treatment during the covid-19 pandemic.

## METHODS

### Ethical aspects

The Research Ethics Committee of a public university approved this study. Because it is an online Informed Consent Form, students who agreed to participate were instructed to click on the “I have read and agree to participate in the survey” button. If they did not agree to participate, they were told to close the page in the browser.

### Design, period and place of study

It is a quasi-experimental study that evaluated a single group before and after the intervention. It was conducted in a federal public university health center in Zona da Mata Mineira from September 2021 to August 2022. In this design, the researchers applied the “auriculotherapy” intervention and observed its effect on the “quality of life” outcome.

The health center where the intervention took place is an outpatient clinic that provides medical, nutritional and dental care, as well as physiotherapy, nursing, laboratory and radiographic examinations for students, teachers and technical-administrative staff.

### Population and sample; criteria of inclusion and exclusion

Students assisted in an extension project, which offers integrative and complementary practices in health for the university community, constituted the population of the present study. The project serves about 60 people per semester.

The study used a convenience sample since it invited all students to collaborate with the research. The study adopted this recruitment strategy since academic activities were gradually resumed due to the pandemic context at the time of data collection. Also, due to the small number of students performing face-to-face activities at the university the researched involved all those who expressed interest.

The inclusion criteria adopted were: students aged 18 years or over and with the availability of time to submit to treatment sessions as well as to respond to the instruments and scales of the study. The study used exclusion criteria for participation in the auriculotherapy intervention: infection, inflammation, injury, or deformity in the ear; use of ear piercing (except for regular earring); use of hearing aids; and being pregnant. Those students who missed two consecutive sessions or more than ten days between them, who reported intense discomfort at the site of application of the seeds and, therefore, decided not to continue the treatment, and who did not fill out the data collection instruments adequately were discontinued from treatment.

Although 70 students met the eligibility criteria and participated in the initial evaluation, 44 completed the intervention protocol (a loss of 37.14%).

### Study protocol

When recruiting students, researchers advertised vacancies for auriculotherapy treatment on the social networks of the extension project. Participants who met the eligibility criteria and agreed to participate in the study signed the Informed Consent form in digital format. Then, they responded to the sociodemographic and clinical characterization instrument and were evaluated for quality of life (initial assessment) through an online form. The study adopted such a strategy to reduce paper manipulation, which could contribute to the spread of the virus. Then, the participants received a schedule to attend the health center for auriculotherapy. At the end of the treatment, they were evaluated again for their quality of life and were asked to report on their satisfaction with the treatment received.

Auriculotherapy was performed with mustard seeds in ten sessions, once a week, alternating the ear at each session, for two months. The acupoints applied were Shenmen, Kidney, Autonomic Nervous System, Heart, Brain Stem, Liver Yangs 1 and 2^(13)^, Liver, Spleen, and Lung 1 and 2. This protocol focuses on managing stress, anxiety, and depression^(13)^ and was selected due to the frequent complaints of emotional changes of university students in the pandemic context.

Before starting the application of seeds, the nurse performed the ear antisepsis with cotton wool and 70% ethyl alcohol. Then, the seeds were adhered to the ear with microporous tape, with the participant seated. Participants were instructed to perform manual pressure on the auricular points at least five times a day^(14)^ and not to remove the seeds until the next session. The intervention was performed by a nurse specialist in acupuncture with more than ten years of experience in the area.

Quality of life, as verified by the World Health Organization Quality of Life-Bref (WHOQOL-Bref)^(3)^, was determined as the primary outcome of the present study. In addition, the study considered the following covariates: sociodemographic and clinical characterization, satisfaction, need for intervention, and general health status after treatment.

The WHOQOL-Bref, developed by the World Health Organization’s quality of Life Group^(4)^, is one of the most used field instruments to assess the quality of life, in addition to the physical and mental health of individuals^(15)^. It consists of 26 questions, being two general questions about the quality of life (perception of quality of life and satisfaction with health) and the others (24 questions) representing each of the 24 facets, divided into four domains^(4)^: Physical domain (pain and discomfort, energy and fatigue, sleep and rest, mobility, the activity of daily living, dependence on medication or treatment, and ability to work); psychological domain (positive feelings, thinking, learning, memory and concentration, self-esteem, body image and appearance, negative feelings, and spirituality/religion/personal beliefs); social relationships domain (personal relationships, social support and sexual activity); and environment domain (physical safety and protection, home environment, financial resources, health and social care [availability and quality], opportunity to acquire new information and skills, participation and opportunities to recreation/leisure, physical environment (pollution, noise, traffic, climate and transport).

Each question has a score from 1 to 5 on a Likert-type scale, which is transformed into a linear scale from 0 (least favorable quality of life) to 100 points (most favorable quality of life)^(4,16)^. The WHOQOL-Bref was translated and validated for the Brazilian version and has adequate psychometric properties^(4)^.

The sociodemographic and clinical characterization instrument, prepared by the study researchers, contained the following variables: gender; age; course; chronic diseases; medications for continuous use; and reason for seeking auriculotherapy.

Participants were asked to rate how the intervention went at the end of treatment. For this, they pointed out, on a scale of 1 to 5, the degree of satisfaction with the performance of the intervention (“extremely dissatisfied;” “dissatisfied;” “not sure;” “satisfied;” “extremely satisfied”), the need to perform the intervention (“totally unnecessary;” “unnecessary;” “not sure;” “necessary;” “totally necessary”) and the general health condition after the end of the treatment (“much better;” “better;” “no change;” “worse;” “much worse”). They also evaluated the presence of adverse reactions resulting from auriculotherapy and their intensity on a scale from 0 (no discomfort) to 10 (intense discomfort).

These instruments underwent refinement by five specialist nurses before data collection, who judged them adequate.

### Analysis of results and statistics

The data collected were analyzed using the statistical software Statistical Package for the Social Sciences, version 23.0. The continuous variables were described using the mean, deviation standard, median, interquartile range, and minimum and maximum values. Absolute and relative frequencies were used to describe categorical variables. The Shapiro-Wilk test was used to verify the data distribution, which showed the non-normal distribution of the scalar variables. The Wilcoxon test was used for paired samples to observe the effect of the intervention on quality of life, considering a 5% significance.

## RESULTS

Of the 44 students who participated in the study, most were women (88.64%), from health courses (90.90%), and did not work (79.50%). Of the total, 36.40% had some chronic disease, and 81.90% used continuous medication. The median age was 22 years (22-24), and the main reason for seeking treatment was emotional changes (90.90%) (Table 1).

**Table 1:** Sociodemographic and clinical characterization and reason for seeking treatment (N = 44), Viçosa, Minas Gerais, Brazil, 2022

Sociodemographic and clinical characteristics	n^*^	%^†^
Sex		
Female	39	88.64
Male	5	11.36
Undergraduate course area		
Health	40	90.90
Exact Sciences	2	4.55
Human Sciences	2	4.55
Chronic disease^‡^		
No	28	63.63
Respiratory	9	20.45
Emotional	3	6.80
Musculoskeletal	2	4.55
Visual	1	2.30
Gastrointestinal	1	2.30
Use of continuous medication^‡^		
No	8	18.18
Hormonal	9	20.40
Psychotropic	22	50.00
Bronchodilator	1	2.30
Corticosteroid	1	2.30
Anticonvulsant	1	2.30
Antihypertensive	1	2.30
Ferritin	1	2.30
Reason for seeking treatment^‡^		
Emotional changes	40	90.90
Musculoskeletal/rheumatic pain	5	11.36
Migraine/Headache	4	9.10
Improvement in academic performance (concentration, focus, energy)	3	6.80
Improved quality of life	3	6.80
Changes in sleep	2	4.55
Respiratory changes	1	2.30

* n - absolute frequency;

† % - Relative frequency;

‡ The participant could mark more than one answer option.

Statistically significant improvement was found in all domains of quality of life after treatment with auriculotherapy (Table 2).

**Table 2:** Quality of life before and after auriculotherapy (N = 44), Viçosa, Minas Gerais, Brazil, 2022

Domains	Pre-intervention (initial assessment)			Post-intervention (final assessment)			*p* value^||^
	Mean (SD^*^)	Median (IR^†^)	Min.^‡^; Max.^§^	Mean (SD^*^)	Median (IR^†^)	Min.^‡^; Max.^§^	
Perception of quality of life	61.93 (20.52)	75.00 (50.00-75.00)	0.00; 100.00	76.70 (13.63)	75.00 (75.00-75.00)	50.00; 100.00	<0.001
Satisfaction with health	44.89 (21.95)	50.00 (25.00-75.00)	0.00; 75.00	61.93 (21.22)	75.00 (50.00-75.00)	0.00; 100.00	<0.001
Physical	55.76 (16.41)	57.14 (46.43-67.86)	25.00; 89.00	71.51 (11.92)	71.43 (65.18-81.25)	39.00; 89.00	<0.001
Psychological	49.05 (14.92)	50.00 (41.67-62.50)	8.00; 79.00	64.49 (10.76)	62.50 (58.33-75.00)	42.00; 83.00	<0.001
Social Relations	60.98 (16.05)	58.33 (50.00-75.00)	25.00; 92.00	74.05 (15.69)	75.00 (66.67-83.33)	42.00; 100.00	<0.001
Environment	59.09 (11.19)	59.38 (53.13-65.63)	28.00; 88.00	70.17 (9.32)	68.75 (65.63-77.34)	53.00; 91.00	<0.001

* SD - standard deviation;

† IR - interquartile range;

‡ Min - Minimum value;

§ Max - Maximum Value;

|| Wilcoxon test for paired samples.

Most of the students (93.20%) were satisfied or extremely satisfied with the treatment performed, considered the intervention necessary or totally necessary (90.90%); and the general health condition after the end of the treatment was reported as better or much better (88.60%), as evidenced in Table 3.

**Table 3:** Satisfaction, need for the intervention, and general health condition (N = 44), Viçosa, Minas Gerais, Brazil, 2022

Variables	n^*^	%^†^
Satisfaction with the intervention		
Extremely dissatisfied	2	4.45
Unsatisfied	0	0.00
Not sure	1	2.30
Satisfied	15	34.10
Extremely satisfied	26	59.10
Need for intervention		
Totally unnecessary	0	0.00
Unnecessary	0	0.00
Not sure	4	9.10
Necessary	17	38.63
Totally necessary	23	52.27
General health condition after completion of the intervention		
Much worse	1	2.30
Worse	1	2.30
No change	3	6.80
Best	25	56.80
Much better	14	31.80

* n - Absolute frequency;

† % - Relative frequency

Finally, 9.10% (n = 4) of the students reported adverse reactions resulting from auriculotherapy, such as pain or discomfort at the site of application of the seeds (6.80%; n = 2; mean intensity = 4/10; standard deviation = 1.41); headache (2.30%; n = 1; intensity = 10/10) and dizziness at the time of application (2.30%; n = 1; intensity = 3/10). The researchers followed these participants and reported the symptoms as punctual, transient, and tolerable.

## DISCUSSION

The present study evidenced that the auriculotherapy protocol adopted could improve the quality of life of university students in the context of the Covid-19 pandemic during the established treatment period. Specifically, there was a 19.65% increase in the perception of quality of life after treatment; and most of the students were satisfied with the intervention performed, found it necessary, and reported a better general health condition after treatment.

These findings are innovative since this is the first investigation that evaluated the action of auriculotherapy in university students in the pandemic context. Such studies like this one are relevant because this population suffered significant mental impacts in this period, aggravating the existing psychological conditions, emergence of new cases, and increased self-medication and suicide ideation/attempt^(17)^. In this context, auriculotherapy can be a support intervention for students in mental suffering, in addition to acting in the prevention of new diseases.

Some studies have already reported beneficial effects of auriculotherapy on mental health, especially anxiety and depression levels in university students, outside pandemic contexts^(18)^. Its effects on quality of life have also been investigated in other populations, such as nursing professionals, especially in the mental aspect^(19)^. However, the neurophysiological pathways to explain the triggering of the effects are the same. Researchers believe the effects may occur through modulation of the hypothalamic-pituitary-adrenal axis^(20)^. With the auricular stimulus, activation of limbic cortical regions occurs^(21)^ and the release of endorphins and enkephalins^(8)^, which will contribute to the reduction of anxiety, stress and depression levels, for example.

The auriculotherapy protocol adopted in this study was based on a systematic review that recommended the use of auricular acupoints Shenmen, Kidney, Autonomic Nervous System, Heart, Brain Stem and Liver Yangs 1 and 2, for control of anxiety, stress and depression in adults^(13)^. Point Shenmen has a sedative action; the Kidney, energetic and invigorating function; the Autonomic Nervous System regulates the functioning of the sympathetic and parasympathetic nervous systems, with an effect on pain and muscle relaxation; The Heart controls blood circulation and mental and emotional activities - it is sedative and relaxing; the Brain Stem has a calming function; and the Liver Yangs 1 and 2 control the rise of Yang of the liver, which is an energy pattern in the face of stress^(13)^. The present investigation also added the spleen points (which is related to excessive worry and is responsible for applied thinking, intelligence, study, memory, concentration and focus), Liver (which controls feelings of irritability and anger and regulates sleep), and Lung (which regulates feelings of sadness)^(22)^.

Mustard seed was also used as a stimulation device, as it is not invasive and consequently increases acceptability by students. Seeds and ear needles were the most used devices in studies included in a systematic review, whose objective was to analyze the principal protocols for the application of the intervention in the treatment of stress, anxiety and depression in adults and the elderly, with an average of 11 sessions^(13)^. However, in the present study, there was considerable withdrawal of treatment (37.1%). It was probably due to the relatively long follow-up time (ten sessions held in two months). Because of this and the superior effects of semi-permanent needles in relation to seeds^(23)^, further studies using invasive devices are suggested to verify the possibility of a shorter follow-up time.

After auriculotherapy, student satisfaction with their health increased by 27.51%, and this increase was statistically significant. When a person reaches satisfactory levels of comfort and well-being, they are able to adopt habits that improve their health and their relationship with their body and mind^(24)^. Therefore, it is an intervention that can help promote the physical and mental health of students.

There was also a statistically significant increase of 22.02% in the physical domain of quality of life, which is related, among other factors, to pain, fatigue, sleep, and drug dependence. These were some of the reasons reported by the students for seeking treatment. The literature presents positive evidence of this intervention concerning pain^(25)^ to sleep^(26)^, and dependence on medication^(27)^. These studies were not conducted with the same population of the present investigation, which highlights the relevance of the results presented here. Also, it is pertinent to point out that it was not evaluated whether there was a reduction in the consumption of medicines. The study did not consider this variable since it investigated only the use of continuous medications, and the follow-up time to verify such behavior was relatively short. Therefore, it is appropriate that studies also evaluate the impact of auriculotherapy on the consumption of psychotropic drugs.

Ninety-nine percent of the students reported complaints of emotional changes to seek treatment, and the Psychological Domain of quality of life was the one with the highest statistically significant increase among those investigated (23.94%). Several studies reported auriculotherapy to reduce psychological signs and symptoms such as anxiety^(14,28)^ and depression^(18)^ in college students. However, no studies were found with the use of this intervention in the pandemic context. In addition, due to the promising results reported here, it is suggested to conduct large randomized clinical trials to allow the generalization of the findings and, consequently, to implement auriculotherapy as a therapy for the promotion and maintenance of mental health of university students.

Social relationships can also be impacted by emotional disorders associated with the psychological domain of quality of life, especially in the context of the Covid-19 pandemic^(29)^. They can help with the overload of stress and anxiety generated by the pandemic. However, restrictive measures and social distancing have prevented these practices^(30)^. From this perspective, this study shows the importance of auriculotherapy as an effective device in the management of social relationships among university students. The results showed an increase of 17.65% in this domain. This finding may be related to the fact that, when receiving care, the student felt welcomed and interacted with the therapist, which can be characterized as social support.

Regarding the Environment domain, the study observed a statistically significant increase of 15.29%. The facets that make up this domain are not modifiable by an internal and individual perspective since they are subject to the collective and society. Therefore, when observing the improvement in the score of this domain, it can be inferred that, by improving the levels of the other domains (perception of quality of life, satisfaction with health, physical, psychological and social relationships), it is possible to modulate feelings of pessimism, which favors the disposition of people to a more positive perspective on the aspects that surround them^(31)^.

Concerning satisfaction with the intervention performed, most of the students were extremely satisfied; that it is totally necessary; and that the perception of their general health condition is better after treatment. In line, a study conducted with war veterans with post-traumatic stress disorder showed that auriculotherapy had high acceptability^(32)^.

In the present study, no participant had severe adverse events. The most reported discomforts were painful sensitivity in the ear, headache, and dizziness. A systematic review of the literature, which included 18 randomized clinical trials of adverse events related to auriculotherapy with a sample of 1,753 people, reported that symptoms of irritation and local discomfort, sensitivity or mild pain, and dizziness were common. However, they were transient, mild, and tolerable, and no serious adverse events were identified, providing evidence that the intervention is relatively safe^(33)^. In addition, it is also a low-cost approach, requiring little time for application and minimally invasive^(13)^.

For future studies, it is relevant to investigate the impact that auriculotherapy may cause, in the medium and long term, on academic performance and school dropout rate; in addition to analyzing the perception and feelings of students about auriculotherapy, in the pandemic or post-pandemic context, through qualitative approaches.

### Study limitations

The recruitment of the sample by convenience is a limitation of the present study that may interfere with the generalization of the findings. However, due to the pandemic context, this strategy was designed to recruit the largest number of students present at the University at that time. The student also highlights the high rate of treatment withdrawal (37.1%); this could have been due to their extended time (two months). In future studies, the study suggests using more effective delivery devices, such as ear needles, which may reduce follow-up time. In addition, the absence of a control group made it impossible to compare with students who did not receive the intervention. However, at that time, the sample was not sufficient to conduct a large clinical trial. Nevertheless, significant results were achieved with this design and sample size.

### Contributions to the field of nursing

The findings of this study contribute to the advancement of scientific knowledge in the area of health and nursing since the implementation of auriculotherapy in the health care services of university students can help promote well-being, quality of life, and mental health, in addition to contributing, in the medium and long term, to improve academic performance and reduce university dropout.

It is an intervention that is easy and quick to apply, as trained therapists perform it. It is low cost, with minimal side effects, and allows mass treatment. In this sense, the study recommends that nurses working in student health care appropriate this technique as another intervention tool to support the quality of life of university students.

## CONCLUSIONS

According to the established protocol, auriculotherapy could improve the perception of Quality of Life, Satisfaction with Health, as well as the Physical, Psychological, Social Relations and Environment domains of WHOQOL-Bref in university students in the context of the Covid-19 pandemic, during the time of treatment. Most of the students were extremely satisfied with the treatment performed, considered the intervention totally necessary, and the general health condition after completion was reported as better.

These results support auriculotherapy as an intervention that can favor the promotion of well-being, quality of life, and mental health of this population. It is recommended that more studies be conducted, particularly randomized clinical trials to investigate the real effectiveness of auriculotherapy in these variables. Thus, it will be possible to safely implement this intervention to promote the quality of life and mental health of the university community.

## SUPPLEMENTARY MATERIAL

https://doi.org/10.48331/scielodata.E5UV58
